# Supplementary material for: The Functional Diversity of the High-Affinity Nitrate Transporter Gene Family in Hexaploid Wheat: Insights from Distinct Expression Profiles
Source: Int J Mol Sci. 2023 Dec 29;25(1):509. doi: 10.3390/ijms25010509 (PMC10779101; doi:10.3390/ijms25010509)
Supplement: Supplementary file 1 [file ijms-25-00509-s001.zip › Supplementary Figures.pdf]

Supplementary Figures

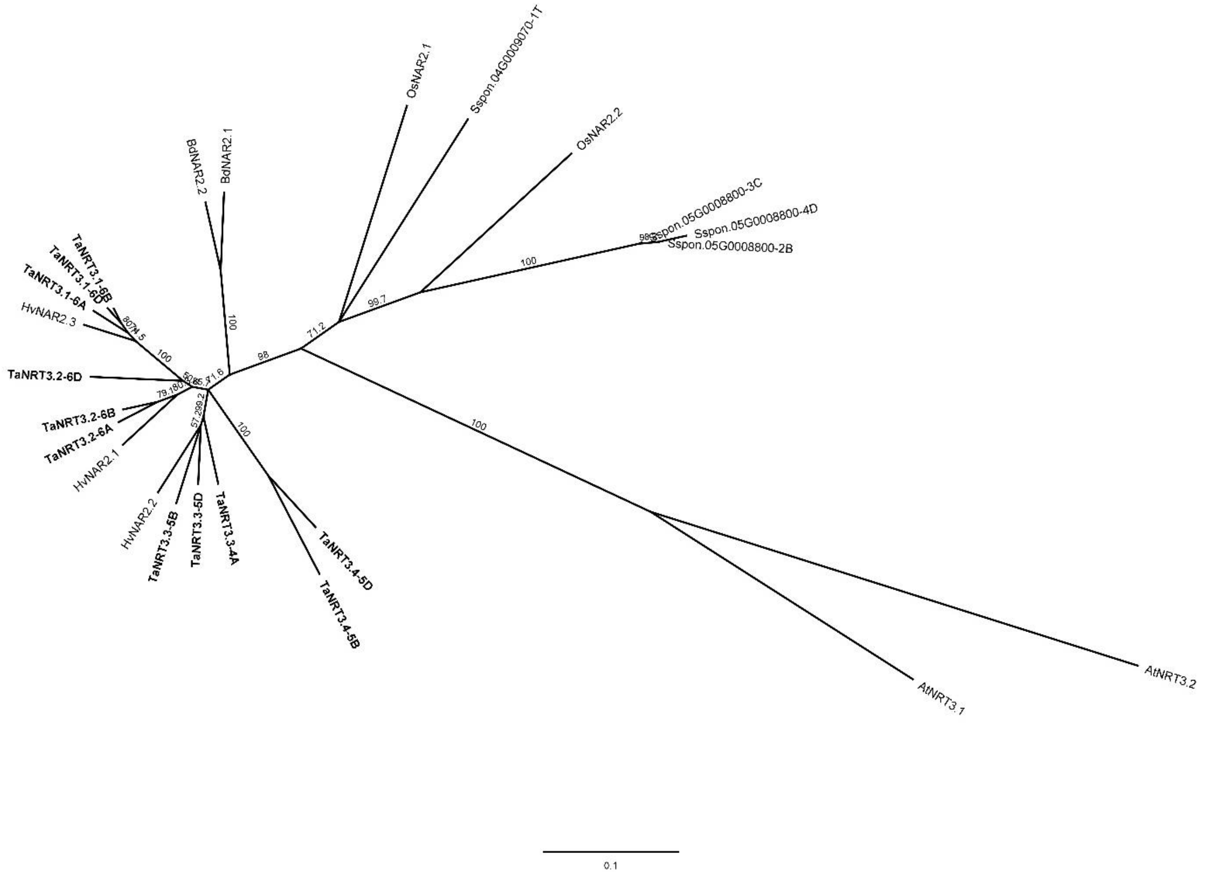

Figure S1. Phylogenetic relationship of *NRT3* genes of hexaploid wheat, rice, barley, sugarcane, Brachypodium, and Arabidopsis. The protein coding DNA sequences plus 200 bp of the 3'-noncoding regions were aligned using MUSCLE sequence alignment and the tree was constructed using the neighbour-joining method. The bootstrap values, expressed as a percentage, were obtained from 1000 replicates. The scale bar corresponds to genetic distance, expressed as number of nucleotide substitutions per site. The accession number of the sequences used in the analysis can be found in Table S1 and the percentage identity matrix can be found in Table S4.

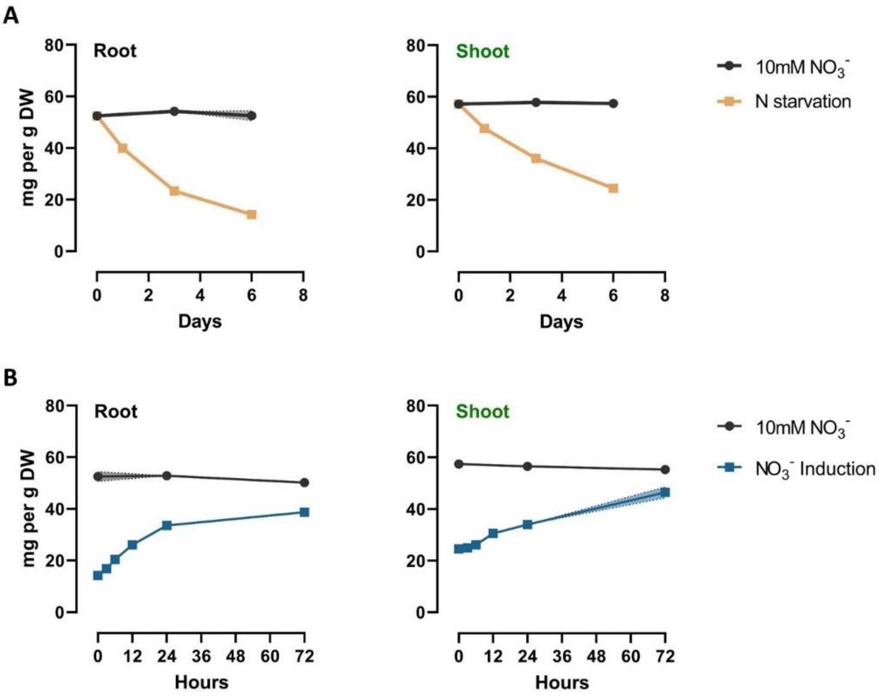

Figure S2. Total N content per dry weight in the root and shoot of wheat (cv Paragon) in response to N starvation and nitrate induction. **(A)** Plants were introduced to N starvation 2 weeks after germination and the response was monitored at different time points after starvation (1, 3, and 6 days after). Fisher's LSD (5%): Root 2.74; Shoot 1.78. **(B)** N-starved plants for 6 days were supplied with 10 mM nitrate and the response was monitored at different time points after nitrate provision (3, 6, 12, 24, and 72 hours after). For comparison data from plants growing in 10 mM nitrate were included. Values are means of three biological replicates and the shaded area corresponds to the standard error bands. Fisher's LSD (5%): Root 2.30; Shoot 2.86.

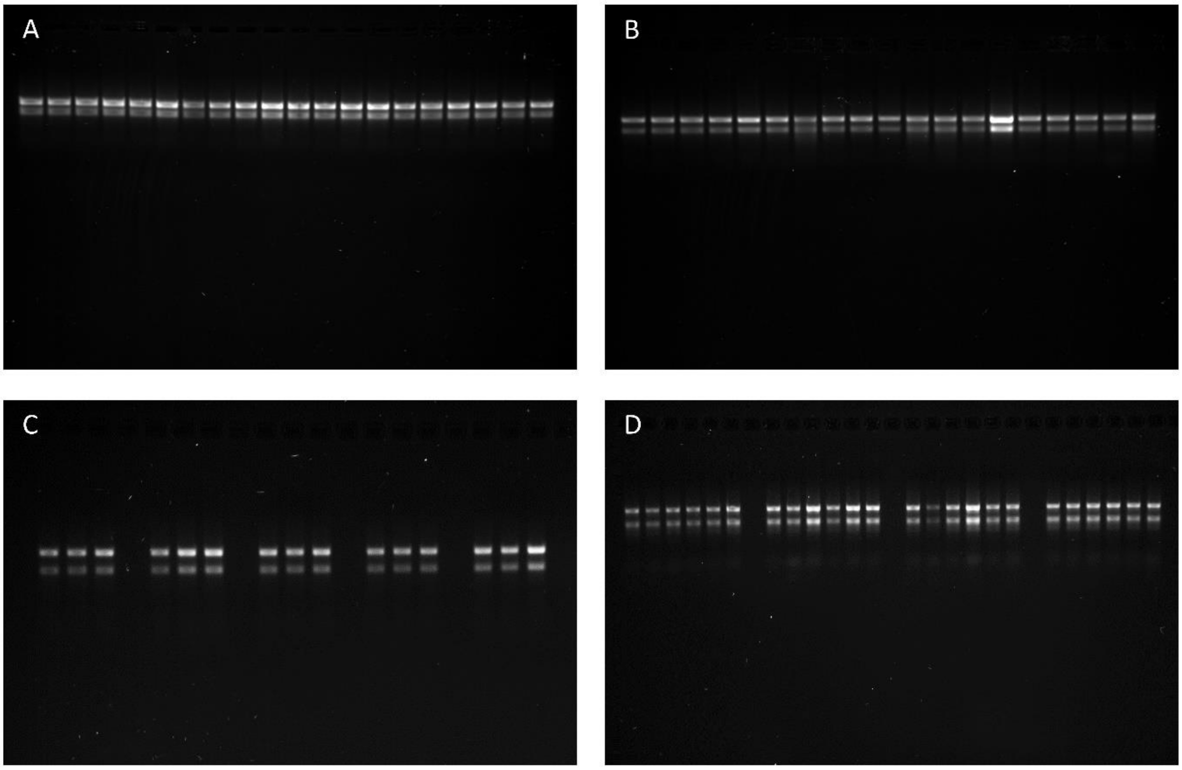

Figure S3. RNA sample quality assessment with gel electrophoresis. Gel electrophoresis of 500 ng total RNA samples used for the expression analysis **(A-B)** in the root and **(C-D)** the shoot of wheat from the N starvation and nitrate induction time course analysis.
